# Consequences of COVID-19 Among Adult HIV Patients Versus Non-HIV Patients: Two-Year Data From the Primary Isolation Centre in Sudan

**DOI:** 10.7759/cureus.36939

**Published:** 2023-03-30

**Authors:** Omer Nemery, Abdelsalam M.A. Nail, Mohammed S Hamed, Ziryab Imad, Jimmy William

**Affiliations:** 1 Infectious Diseases, Tropical Diseases Teaching Hospital - Voluntary Counseling and Testing and Anti-retroviral Therapy Centre (Omdurman), Omdurman, SDN; 2 Internal Medicine, Omdurman Islamic University, Omdurman, SDN; 3 Internal Medicine, Sudan Medical Specialization Board, Khartoum, SDN; 4 Internal Medicine, Haj Elsafee Hospital, Khartoum North, SDN; 5 Internal Medicine, University of Bahri, Khartoum, SDN; 6 Internal Medicine, Ziryab Research Group, Khartoum, SDN; 7 Internal Medicine, Sligo University Hospital, Sligo, IRL

**Keywords:** sudan, isolation, sars-cov-2, covid-19, hiv/aids

## Abstract

Background

The COVID-19 pandemic remains to have a global impact despite the great efforts in prevention. Controversy persists regarding the outcomes of SARS-CoV-2 among HIV patients versus non-HIV individuals.

Objective

This study aimed to assess the impact of COVID-19 among adult patients with HIV versus non-HIV in the chief isolation centre in Khartoum state, Sudan.

Methods

This is an analytical cross-sectional, comparative single-centre study conducted at the Chief Sudanese Coronavirus Isolation Centre in Khartoum from March 2020 to July 2022. Data were analysed using SPSS V.26 (IBM Corp., Armonk, USA).

Results

This study included 99 participants. The overall age mean was 50±1 years old, with a male predominance of 66.7% (n=66). 9.1% (n=9) of the participants were HIV cases, 33.3% of whom were newly diagnosed. The majority, 77.8%, reported poor adherence to anti-retroviral therapy. The most common complications included acute respiratory failure (ARF) and multiple organ failure, 20.2% and 17.2%, respectively. The overall complications were higher among HIV cases than non-HIV cases; however, statistically insignificant (p>0.05 ), except for acute respiratory failure (p<0.05). 48.5% of participants were admitted to the intensive care unit (ICU), with slightly higher rates among HIV cases; however, this was statistically insignificant (p=0.656). Regarding the outcome, 36.4% (n=36) recovered and were discharged. Although a higher mortality rate was reported among HIV cases compared to non-HIV cases (55% vs 40%), it was statistically insignificant (p=0.238).

Conclusion

The mortality and morbidity percent proportion among HIV patients with superimposed COVID-19 infection was higher than in non-HIV patients but statistically insignificant aside from ARF. Consequently, this category of patients, to a large extent, should not be considered highly susceptible to adverse outcomes when infected with COVID-19; however, ARF should be closely monitored for.

## Introduction

The coronavirus disease (COVID-19) is caused by a novel coronavirus, Severe Acute Respiratory Syndrome Coronavirus 2 (SARS-CoV-2), affecting mainly the respiratory system; however, it has several implications [1]. It has the worrisome characteristics of rapidly spreading among communities, causing a severe disease pattern, particularly among the elderly and those with underlying comorbidities [2]. Respiratory droplets are the main human-to-human transmission route, whether through coughing or sneezing [2]. Transmission from contaminated surfaces is well established [3]. SARS-CoV-2 has an average incubation period of 5-6 days, extending to two weeks, with infected individuals being asymptomatic for several days [2,3]. COVID-19 clinical manifestations are not specific, extending from asymptomatic to severe symptoms such as flu-like symptoms only to Acute Respiratory Distress Syndrome (ARDS) and multiple organ failure causing fatality [4]. Common symptoms include anosmia, sore throat, dry or wet cough, fever, myalgia, and dyspnea [2, 4].

COVID-19 has impacted universal health systems, financial systems, and communities and has caused substantial morbidity and mortality rates worldwide [4]. Globally, 472,816,657 COVID-19 cases were confirmed as on the 23rd of March 2022, including 6,099,380 deaths reported to WHO. As of the 17th of March 2022, 10,925,055,390 vaccine doses have been administered [5]. 13th of March 2020 marked the first positive COVID-19 case reported in Sudan; however, figures reached 61,862 positive cases reported to the WHO by the 24th of March 2022, including 4,900 deaths. As of the 6th of March 2022, 6,131,070 vaccine doses have been administered in Sudan [6]. The global case fatality rate was 6.7% [7]. Patients with obesity, diabetes, and hypertension were more likely to die, be admitted to ICUs, and have a more severe form of infection. Nevertheless, there are conflicting reports concerning the co-infection of Human Immunodeficiency Virus (HIV) patients with COVID-19 [8]. 

Sub-Saharan Africa exhibits the utmost burden of HIV and patients not receiving anti-retroviral therapy (ART) [9]. Currently, 40 million individuals coexist with HIV globally, of which 26 million dwell in Africa, with a substantial percentage not receiving ART, compared to elsewhere worldwide [10]. HIV results in immunosuppression by depleting CD4+ cells, reducing the capacity of the immune cells to defend against various pathogenic infections such as COVID-19 [11]. The risk of developing a rather severe pattern of infections, in general, is higher when immunosuppressed and even more remarkable when not receiving ART [12]. The impact of the COVID-19 pandemic on a rather significant number of individuals with HIV globally is very challenging for health systems worldwide, as more aggressive preventive and therapeutic measures might be required for this population [12, 13]. Therefore, several studies explored the implications, hospitalisation risk, and outcomes attributed to HIV in patients with COVID-19 co-infection; however, results were primarily conflicting [14-18]. Therefore, our study aimed to explore the implications of COVID-19 among HIV patients in contrast to non-HIV patients admitted to the primary isolation centre in Khartoum state, Sudan, over two years to reach concise outcomes based on which guidelines on managing HIV/COVID-19 co-infections can be implemented.

## Materials and methods

This is a hospital-based comparative study conducted in Khartoum state, Sudan, in 2020-2022 to assess the impact of COVID-19 among adult patients with HIV versus non-HIV at the primary isolation centre (Jabra Hospital) in Khartoum state, Sudan. The study was approved by the Institutional Review Committee (IRC) at Jabra Hospital. Data were collected by reviewing all cases admitted in this centre from March 2020 to July 2022 by trained registrars. Detailed demographic characteristics, clinical characteristics, aetiology, HIV infection characteristics, and COVID-19 outcome assessment were obtained after informant consent. Diagnosis of HIV was confirmed by enzyme-linked immunosorbent assay (ELISA), and COVID-19 infection by nasopharyngeal swab reverse transcription polymerase chain reaction (RT-PCR). Data were entered into a computer database. SPSS version 26 was used for analysis.

Primary isolation centre

Jabra Hospital was the primary/first isolation centre in Khartoum State, Sudan, with a capacity of almost 90 beds. In addition to COVID-19 patients, all HIV cases with COVID-19 co-infection were transferred from all other isolation centres in Khartoum state to Jabra Hospital.

Study population and selection

The study population included all adult patients (≥ 18 years old) with COVID-19 admitted to Jabra Hospital for isolation. All HIV patients (Exposed group) that fulfilled the inclusion criteria were enrolled. Non-HIV patients fulfilling the inclusion criteria (Unexposed-control group) were randomly selected (in a ratio of 1:10).

Inclusion Criteria: 1. All patients who were admitted for isolation during the study period; 2. Patients with complete medical records (severity status, complication developed, etc.); and 3. Patients who completed their isolation period.

Exclusion Criteria: 1. Refusal to participate; and 2. Patients with incomplete record charts.

Other specific variables classifications include the following:

1. COVID-19 severity was classified into Mild, Moderate, and Severe based on WHO Criteria [19]: Mild: Symptomatic adult patients meeting the case definition for COVID-19 without evidence of viral pneumonia or hypoxia; Moderate: Adult patients with clinical signs of pneumonia (fever, cough, dyspnoea, fast breathing) but no signs of severe pneumonia, including SpO2 ≥ 90% on room air; Severe: Adult patients with clinical signs of pneumonia (fever, cough, dyspnoea, fast breathing) plus one of the following: respiratory rate >30 breaths/min; severe respiratory distress; or SpO2 < 90% on room air with clinical signs of pneumonia (fever, cough, dyspnoea, fast breathing) plus one of the following: respiratory rate >30 breaths/min; severe respiratory distress; or SpO2 < 90% on room air. 2. Socio-economic status was classified into Low, Middle, and High based on Modified BG Prasad Socio-economic Classification [20]. This scale is based on the per-capita monthly income (per-capita monthly income = total monthly family income/total family members) and therefore, applies to individuals. 3. Poor adherence to ART was defined as adherence of <90% of the prescribed dose [21].

## Results

**Figure 1:**
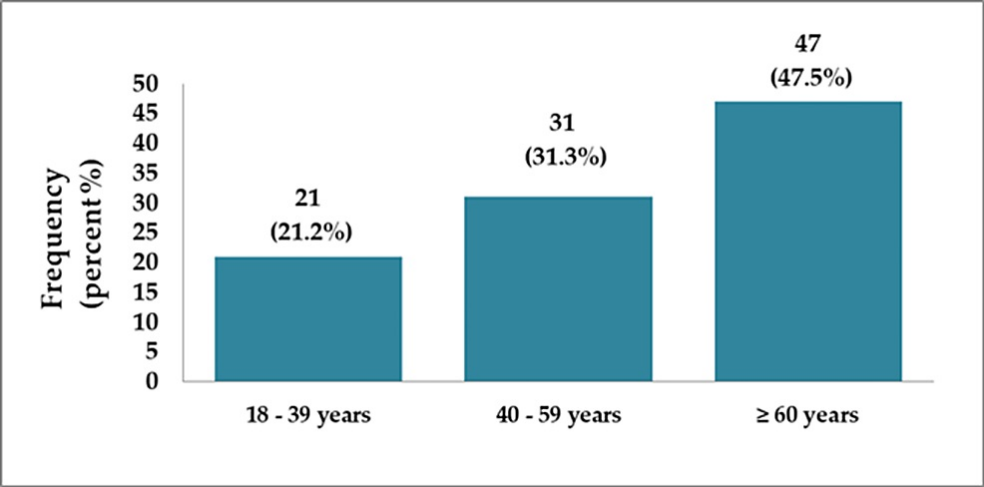
Age distribution among participants

**Figure 2:**
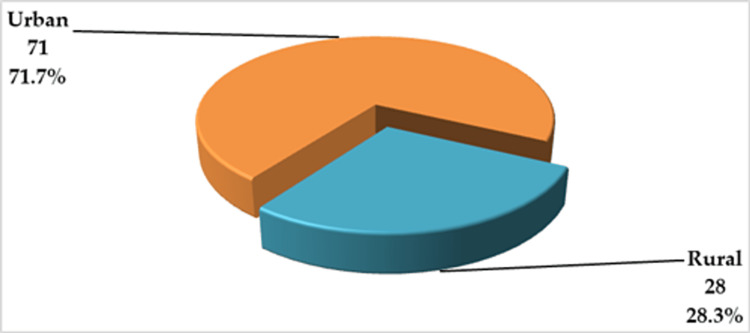
Residence distribution among the study population

This study included 99 COVID-19 patients admitted to the Jabra Hospital for isolation in Khartoum state, Sudan, during the study period. The overall age mean was 50±1 years, with nearly half, 47 (47.5%), being older than sixty years in age (Figure 1), with male gender dominance of 66 (66.7%) and a ratio of 2:1 (Table 1). In addition, 71 (71.7%) were from urban residential areas (Figure 2), and the majority, 85 (85.9%), were of low socioeconomic class (Table 2). Among both study groups, statistical significance testing (via Fischer Exact test) on variables: Age, Gender, and Residence were found to be insignificant (p-value > 0.05). Nevertheless, the p-value was <0.05 among patients with low socioeconomic status and the HIV-positive case group.

**Table 1:** Gender distribution among participants

Gender	Frequency	Percent (%)
Male	66	66.7
Female	33	33.3
Total	99	100

**Table 2:** Socioeconomic status of the study population

Socioeconomic class	Frequency	Percent (%)
Low	85	85.9
Middle	11	11.1
High	3	3.0
Total	99	100

**Figure 3:**
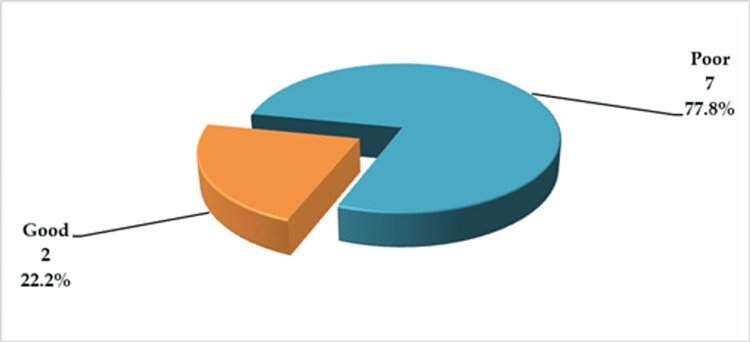
Adherence to HIV therapy among the study participants

The most commonly reported comorbidities were Hypertension 32 (32.3%), Diabetes 12 (12.1%), and Multiple Comorbidities 32 (32.3%); however, no statistically significant difference was identified between both study groups (Table 3). More than half of the participants had a moderate infection; 54 (54.5%) and 45 (45.5%) had a severe infection. In this study, nine (9.1%) participants were known HIV cases, of which four (44.4%) were diagnosed from more than 5 years. Moreover, three (33.3%) of them were newly diagnosed. Nevertheless, seven (77.8%) reported poor adherence to anti-retroviral therapy (Figure 3). 

**Table 3:** Comorbidities among participants *The overall Fischer exact test p-value = 0.67; **Multiple Comorbidities included patients with more than one underlying illness; ^Included: hepatitis, asthma, cancers, heart diseases, epilepsy, tuberculosis, renal failure, sickle cell anemia, and stroke

Comorbidities*	HIV Status					
	Positive		Negative		Total	
	Freq.	%	Freq.	%	Freq.	%
Multiple**	2	22.2%	30	33%	32	32.3%
Hypertension	0	0%	11	12.2%	11	11.1%
Diabetes Mellitus	1	11.1 %	11	12.2%	12	12.1%
Others^	2	22.2%	11	12.2%	13	13.1%
None	4	44%	27	30%	31	31.3%
Total	9	100%	90	100%	99	100%

In regards to the outcome, 36 (36.4%) recovered and were discharged in good condition, 22 (22.2%) developed complications, and the mortality rate was 41 (41.8%). Our study showed that the most common complications included acute respiratory failure 20 (20.2%), sepsis, septic shock and multiple organ failure 17 (17.2%), coagulopathy, such as Disseminated Intravascular Coagulopathy (DIC) and Venous Thrombo Embolism (VTE) 15 (15.2%), and cardiovascular complications 11 (11.1%). Nearly half of the participants, 48 (48.5%), were admitted to the intensive care unit. Of those enrolled, 50 (50.5%) were admitted to the isolation centre for less than one week.

Further cross-tabulations were performed to assess the hypothesis associating HIV infection, coronavirus infection severity, and the overall outcome, using the Fischer Exact Test instead of the Chi-square test, given that the observed counts were of small size. Analysis elaborated that the participants who were HIV positive had no significant difference concerning coronavirus disease severity vs non-HIV patients (p=0.727) (Table 4). Moreover, admission to the ICU was slightly higher among HIV cases than non-HIV cases, but the difference was not statistically significant (p=0.727) (Table 5). No significant difference (p=0.360) was reported in the admission duration relative to HIV status (Table 6). Furthermore, higher mortality rates were reported among HIV cases compared to non-HIV cases; nonetheless, the difference was not statistically significant (p=0.238) (Table 7). Also, complications such as sepsis and cardiovascular entities were higher in HIV cases than others, but the difference was insignificant (p > 0.05). However, ARF was significantly higher among HIV cases (Table 8).

**Table 4:** COVID-19 infection severity versus HIV Status *The overall Fischer Exact Test p-value = 0.727

Covid-19 infection Severity*	HIV Status					
	Positive		Negative		Total	
	Freq.	%	Freq.	%	Freq.	%
Moderate	4	44.4 %	50	55.6 %	54	54.5 %
Severe	5	55.6 %	40	44.4 %	45	45.5 %
Total	9	100 %	90	100 %	99	100 %

**Table 5:** HIV status and Intensive Care Unit admission *The overall Fischer Exact Test p-value = 0.735

Admitted to Intensive Care Unit *	HIV Status					
	Positive		Negative		Total	
	Freq.	%	Freq.	%	Freq.	%
Yes	5	55.6 %	43	47.8 %	48	48.5 %
No	4	44.4 %	47	52.2 %	51	51.5 %
Total	9	100.0 %	90	100.0 %	99	100.0 %

**Table 6:** HIV status and isolation/admission period among the study population *The overall Fischer Exact Test p-value = 0.360

Duration of stay in the isolation centre*	HIV Status*					
	Positive		Negative		Total	
	Freq.	%	Freq.	%	Freq.	%
< 1 week	4	44.4 %	46	51.1 %	50	50.5 %
1 - 3 weeks	3	33.3 %	37	41.1 %	40	40.4 %
4 weeks or more	2	22.2 %	7	7.8 %	9	9.1 %
Total	9	100 %	90	100 %	99	100 %

**Table 7:** HIV status and the overall outcome *The overall Fischer Exact test p-value = 0.238

Overall outcome*	HIV Status*					
	Positive		Negative		Total	
	Freq.	%	Freq.	%	Freq.	%
Recovered	1	11.1 %	35	38.9 %	36	36.4 %
Complications	3	33.3 %	19	21.1 %	22	22.2 %
Dead	5	55.6 %	36	40.0 %	41	41.4 %
Total	9	100 %	90	100 %	99	100 %

**Table 8:** HIV status and complications observed during the study period *Fischer Exact Test was performed

Complications		HIV Status						p-value*
		Yes (n = 9)		No (n = 90)		Total (n =99)		
		Freq.	%	Freq.	%	Fre q.	%	
Acute respiratory failure	Yes	4	44.4 %	16	17.8 %	20	20.2 %	0.0001
	No	5	55.6 %	74	82.2 %	79	79.8 %	
Sepsis, septic shock & multiple organ failure	Yes	3	33.3 %	14	15. 6 %	17	17.2 %	0.18
	No	6	66.7 %	76	84.4 %	82	82.8 %	
Coagulopathy	Yes	0	0.0 %	15	16.7 %	15	15.2 %	0.18
	No	9	100 %	75	83.3 %	84	84.8 %	
Cardiovascular complications	Yes	2	22.2 %	9	10.0 %	11	11.1 %	0.26
	No	7	77.8 %	81	90.0 %	88	88.9 %	
Necrotising pneumonia	Yes	0	0.0 %	5	5.6 %	5	5.1 %	0.97
	No	9	100 %	85	94.4 %	94	94.9 %	
Massive pulmonary embolism	Yes	0	0.0 %	2	2.2 %	2	2 %	0.98
	No	9	100 %	88	97.8 %	97	98 %	

## Discussion

COVID-19 and HIV are serious infections associated with higher mortality and morbidity rates than other viral infections. Our study aimed to assess and explore HIV/COVID-19 co-infection among 99 patients admitted with COVID-19 into the Primary Isolation Centre (Jabra Hospital) in Khartoum state, Sudan, from 2020-2022. In this study, the overall mean age was 50±1 years; however, the mean age among HIV patients was 44±1 years. 47.5% (n=47) were older than 60 years, similar to the results by Omar et al., who reported that the mean age in Sudan, in general, is 62 (55-70) years old [22]. However, in China, Zheng et al. reported that the mean age of COVID-19 patients was 45 years (range 33.5-57 years) [23], while Lian et al. reported higher age ranges with a corresponding mean age of 68.28±7.31 years [24]. In Jordan, Samrah et al. reported that the mean age was 40 years [25]. Age has independent prognostic significance and may aid in shared decision-making in COVID-19 patients. Furthermore, our study reported a male gender dominance of 66 (66.7%) and a male: female ratio of 2:1. Conversely, in China, Zheng et al. reported that male patients accounted for 49.7% [23]. Furthermore, Lian et al. stated that there was a significantly higher frequency of women in the older patient group than in younger patients (57.35% vs 46.47%, P = .021) [24]. Samrah et al. from Jordan illustrated that about half of the patients (44 [54.3%]) were females, unlike Wan et al., who concluded no significant gender difference, with 53.3% men [25, 26].

The majority of patients, 68 (68.7%), had multiple comorbidities, including predominantly diabetes, hypertension and cardiovascular diseases, followed by isolated diabetes in 12 (12.1%) and hypertension in 11 (11.1%). The prevalence of comorbidities among both study groups was analogous, with an overall p-value of 0.67, thus statistically insignificant. Similarly, Ssentongo et al. [27] concluded that these comorbidities are also prevalent among patients with HIV/AIDS and may increase the adverse effects of COVID-19. Nevertheless, Wan et al. reported a lower prevalence of primary hypertension, diabetes, cardiovascular disease, and malignancy - 9.6%, 8.9%, 5.2%, and 3.0, respectively [26]. Likewise, in Jordan, Samrah et al. reported only one-third (31%) had chronic diseases [25]. Sanyaolu et al. argued that COVID-19 patients with comorbidities, such as hypertension or diabetes mellitus, are more liable to develop a more severe disease course [8]. Furthermore, Sanyaolu et al. observed that elderly patients with comorbidities with COVID-19 infection had increased ICU admissions and mortality rates [8]. Only nine (9.1%) participants had known HIV status; undoubtedly, it is a pre-determined proportion and not a true prevalence. However, our current results are higher than the results published from Wuhan (<1%) previously [28]. Therefore, accurately establishing the incidence of COVID-19 infection among patients with HIV/AIDS versus the general population was unfeasible. Unfortunately, the viral load data were not documented due to a lack of resources and disarray, particularly during the first year of the pandemic. Nonetheless, the majority, seven (77.8%), reported poor adherence to anti-retroviral therapy. Studies reported that several factors in HIV play a significant role during a superimposed COVID-19 infection; CD4 count, viral load, and compliance to ART remain crucial for prognosis and overall outcome [29]. Non-adherence to ART therapy is associated with low CD4 count, high viral load, and progression to AIDS and death [30]. Also, Tesoriero et al. reported that HIV patients with high viral load had an increased risk of hospitalization when co-infected with COVID-19. Nonetheless, several anti-retroviral therapies were suggested to have anti-COVID-19 characteristics; however, the results were ambiguous [31].

On the other hand, among non-HIV patients, a higher overall mortality rate was observed, which could be explained by several factors, including age-related comorbidities, given that the mean age in this group (Control group) was 50±1 years versus 44±1 years among HIV patients (Exposed group). Additionally, complications related to COVID-19 were more pronounced among the control group (Table 7). However, Acute Respiratory Failure was more prevalent among HIV patients than in the control group (44.4% vs 17.8%) and was statistically significant (p=0.001). This is remarkably contrary to several studies [32, 33]; nevertheless, several results, including a global study that included almost 200,000 participants from 38 countries with approximately 17,000 HIV-positive patients, concluded that HIV-positive patients had a 15% more chance of being hospitalized with severe COVID-19, and furthermore, once inpatient, they had a 38% higher mortality than non-HIV patients [17, 34]. 

Nonetheless, a higher mortality percent proportion was noticed among HIV cases than non-HIV cases (5/9 [55.6%] vs 36/90 [40%]); the difference was not statistically significant (p=0.238). Karmen-Tuohy et al. concluded similar findings, stating that HIV insignificantly impacted clinical outcomes in patients with superimposed COVID-19 [33]. Another New York City (NYC) study revealed that patients with HIV/COVID-19 co-infection, almost 30% required mechanical ventilation and roughly 20% died or were discharged to hospice [35]. However, Karmen et al. concluded that both study cohorts (HIV vs Non-HIV) had lower rates of mechanical ventilation requirement yet higher death rates (23.8% vs 11.9% and 28.6% vs 23.8%, respectively), in contrast to the general hospitalized population [33]. Furthermore, another study from NYC reported a lower mortality rate of 21% among hospitalized cases [36]. Two more studies reported that, although mortality rates among HIV patients co-infected by COVID-19 are lower than the general population, a quarter had severe disease, with half of which required ICU admission [37, 38]. Thus, there is controversy regarding mortality and morbidity among HIV patients.

In our current study, nearly half of the participants, 36 (36.4%), recovered and were discharged in good condition, and the mortality rate was 41 (41.8%). The mortality rate was higher than the overall mortality for Sudan currently. According to Altayb et al., the case fatality rate in Sudan was 6.23% [7]. However, this was contrary to Omar et al., who reported that the general mortality rate was 37.5% [22]. Furthermore, Goyal et al. reported only a 10.2% mortality rate (n=40) [30]. Also, in Saudi Arabia, Alyami et al. found that the mortality rate decreased by 6.4% during the study period [39]. Raker et al. elaborated on these findings by the fact that the death census due to COVID-19 does not out into account the collateral impact of the pandemic on mortality rates [40]. Sher et al. added that the collateral impact includes chiefly reduced health service accessibility and psychological repercussion [29]. Another study has emphasised that pandemic-related increased rates of anxiety, low mood and addiction could result in suicide [41]. Another implication would be the pandemic-related global economic crisis, accommodation insecurity and anxiety related to the medical supplies' inaccessibility, particularly in patients with comorbidities [42]. Consequently, the term ''Mortality Excess'' was designed to distinguish between COVID-19-related mortality versus deaths anticipated without; nevertheless, this could be extremely unamenable to assess occasionally [43]. 

Lastly, the main limitation of our study is the relatively limited number of study participants; nevertheless, clear inclusion criteria were followed to ensure outcomes quality. Another limitation was the follow-up, as some outcomes - such as a long-term outcome or the presence of long-term complication - may have required follow-up over a more extended period among HIV patients. So, a long-term prospective cohort follow-up design may be helpful for a more detailed description of the practices that may control complications among discharged patients. Furthermore, the lack of documentation in patient records, such as viral load results, where the research was conducted, and relying on a minor manual system instead of an electronic one has prohibited assessment of other entities.

## Conclusions

This is the first report on HIV/COVID-19 co-infection from the National Isolation Centre in Sudan and one of the few studies with a 2-years follow-up period. In general, morbidity and mortality percent proportion due to COVID-19 among HIV patients was slightly higher than that among non-HIV patients; however, it is statistically insignificant except for ARF. Based on our results, this category of patients should not be considered highly susceptible to adverse outcomes when infected with COVID-19; however, ARF should be closely monitored for. Given the conflicting data regarding this entity, our current study provides substantial outcomes.
